# Epigenetic Silencing of TFPI-2 in Canine Diffuse Large B-Cell Lymphoma

**DOI:** 10.1371/journal.pone.0092707

**Published:** 2014-04-02

**Authors:** Serena Ferraresso, Silvia Bresolin, Arianna Aricò, Stefano Comazzi, Maria Elena Gelain, Fulvio Riondato, Luca Bargelloni, Laura Marconato, Geertruy te Kronnie, Luca Aresu

**Affiliations:** 1 Department of Comparative Biomedicine and Food Science, University of Padova, Padova, Italy; 2 Department of Women’s and Children’s Health, University of Padova, Padova, Italy; 3 Department of Animal Pathology Hygiene and Veterinary Public Health, University of Milano, Milano, Italy; 4 Department Veterinary Science, University of Torino, Torino, Italy; 5 Centro Oncologico Veterinario, Sasso Marconi, Italia; Queen’s University Belfast, United Kingdom

## Abstract

Epigenetic modifications are important early events during carcinogenesis. In particular, hypermethylation of CpG islands in the promoter region of tumor suppressor genes is a well-known mechanism of gene silencing that contributes to cancer development and progression. Tissue factor pathway inhibitor 2 (TFPI-2) is a tumor suppressor involved in invasiveness inhibition. Although TFPI-2 transcriptional silencing, through promoter hypermethylation, has been widely reported in several human malignancies, it has never been explored in lymphoma. In the present study TFPI-2 methylation and gene expression have been investigated in canine Diffuse Large B-cell lymphomas (cDLBCL). The methylation level of 23 CpGs located within the TFPI-2 promoter was investigated by bisulfite-specific PCR and next generation amplicon deep sequencing (GS Junior 454, Roche) in 22 cDLBCLs and 9 controls. For the same specimens, TFPI-2 gene expression was assessed by means of Real-time RT-PCR. Sequence analysis clearly demonstrated that TFPI2 is frequently hypermethylated in cDLBCL. Hypermethylation of the TFPI-2 promoter was found in 77% of DLBCLs (17 out of 22) and in one normal lymph node. Globally, dogs with DLBCL showed a mean methylation level significantly increased compared to controls (p<0.01) and analysis of hypermethylation by site identified 19 loci out of 23 (82%) with mean methylation levels from 2- to 120-fold higher in cDLBCL. Gene expression analysis confirmed a significant down-regulation of TFPI-2 (p<0.05) in DLBCLs compared with normal lymph nodes, suggesting that TFPI-2 hypermethylation negatively regulates its transcription. In addition, a significant positive correlation (p<0.01) was found between TFPI-2 methylation levels and age providing the first indication of age-associated epigenetic modifications in canine DLBCL. To conclude, our findings demonstrated that epigenetic dysregulation of TFPI-2, leading to its reduced expression, is frequently detected in canine DLBCL. In the next future, the aberrant TFPI-2 promoter hypermethylation may be considered in association with prognosis and therapy.

## Introduction

Canine diffuse large B-cell lymphoma (DLBCL) is an aggressive malignancy of the mature B-lymphocytes showing significant overlaps with the human disease and it is considered curable in less than 10% of dogs. Although advances in treatment have improved outcome, survival time is quite variable, ranging from months to years [1]. This incomplete success indicates the need for further studies to elucidate the pathogenesis through the identification of altered gene expression and cellular pathways, thereby influencing the aggressive behavior of DLBCL, and representing a target for novel therapeutic strategies.

In human DLBCL, gene-expression profiling has identified two biologically and clinically distinct molecular subtypes, which consistently correlate with treatment response, regardless of the regimen used ([2] for a review). Nevertheless, paucity remains in the understanding of the molecular events underlying the development and progression of DLBCL, especially in terms of activation of oncogenes or inactivation of tumor suppressor genes (TSG). Recently, it has become clear that, in addition to genetic changes (i.e. mutations, rearrangements), epigenetic modifications represent an important step in the pathogenesis of lymphoma, and such changes may be of interest in the definition of prognosis and delineation of specific therapy [3]. A common epigenetic event is DNA methylation at cytosine residues of CpG islands [4]. CpG islands are short CpG-rich regions, often mapping at the promoter and exonic regions of the gene. Increased methylation of CpG islands within gene promoters is associated with transcriptional gene silencing. In particular, hypermethylation of CpG islands in the promoter region of TSGs is a well-known mechanism of gene silencing that contributes to cancer development and progression [5]. Identifying aberrant methylated genes may provide better understanding of the pathogenesis of lymphoma, thereby paving the way for the development of novel tumor markers and therapeutic targets.

A candidate gene for epigenetic regulation of lymphomagenesis is Tissue Factor Pathway Inhibitor-2 (TFPI-2), a Kunitz-type serine proteinase inhibitor that directly regulates the activation of matrix metallo-proteinases (MMPs) and plays a significant role in the regulation of ECM degradation, which is an essential step for cell remodeling, as well as tumor invasiveness and metastasis. In humans, TFPI-2 is inactivated or absent during tumor progression, conferring an important role for this gene in the pathogenesis of malignancies [6]. Aberrant methylation has been suggested as a possible mechanism for loss of TFPI-2 expression in several human cancers [7]–[11].

We recently characterized the transcriptome profiles of twenty-one dogs with DLBCL and five normal lymph nodes by using an array–based approach [12]. Statistical analyses identified two molecular subtypes similarly to human DLBCL. These two clusters revealed distinct signatures with NFKB2 and other genes related to proliferation having increased expression in the main subgroup. Two probe sets revealed a significant down-regulation of TFPI-2 mRNA levels in both DLBCL groups compared to healthy controls.

The role of TFPI-2 in hematopoietic malignancies is scarcely documented. Epigenetic dysregulation of TFPI-2, leading to its reduced expression, has been observed in pediatric acute myeloid leukemia [13], while no information is available for lymphoma. Major improvements in sequencing technologies have now provided an unprecedented opportunity to examine the cancer-associated genomic alterations even in non-model species.

The aim of the present study was to investigate the TFPI-2 epigenetic status in lymph nodes from dogs with DLBCL and healthy controls. TFPI-2 methylation levels were quantified by means of bisulfite-specific PCR and next generation amplicon deep sequencing. Additionally, the methylation status of TFPI-2 promoter has been correlated to gene expression levels in order to evaluate whether abnormal methylation of the promoter region might be responsible for TFPI-2 transcription silencing.

## Materials and Methods

### Samples and Nucleic Acid Extraction

All lymphoma samples were taken as part of normal diagnostic procedures from dogs with newly diagnosed, previously untreated lymphoma. Only dogs with confirmed DLBCL after histopathological and immunohistochemical analysis (CD20/CD79 marker analysis) were enrolled (Table 1). The study was approved by the University Committee (protocol 20085MSFH2) and a mandatory written consent from patient owners was also requested. Portion of the tumour was obtained under sterile conditions. Normal lymph node samples were obtained from healthy dogs.

**Table 1:** Samples included in the study.

	ID	Breed	Age	Gender	Stage	Substage
**Healthy ctrls**	Ctrl1	German shepherd	5	M		
	Ctrl2	Cane Corso	5	F		
	Ctrl3	Mixed breed	6	M		
	Ctrl4	Mixed breed	4	M		
	Ctrl5	Siberian husky	9	F		
	Ctrl6	Mixed breed	10	F		
	Ctrl7	Mixed breed	9	F		
	Ctrl8	American Pittbul	7	M		
	Ctrl9	Mixed breed	2	F		
**DLBCL**	DLBCL1	Mixed breed	14	F	5	a
	DLBCL2	Scottish terrier	10	F	4	a
	DLBCL3	Pyrenean sheepdog	12	M	3	a
	DLBCL4	English setter	12	F	3	a
	DLBCL5	Labrabor retriever	5	M	4	a
	DLBCL6	Labrador retriever	6	F	4	a
	DLBCL7	Mixed breed	7	F	3	a
	DLBCL8	German shepherd	8	M	3	a
	DLBCL9	Tibetian terrier	8	F	4	a
	DLBCL10	Mixed breed	3	F	3	a
	DLBCL11	Mixed breed	9	M	4	a
	DLBCL12	Epagneul Breton	7	F	5	b
	DLBCL13	Yorkshire terrier	6	M	5	b
	DLBCL14	Mixed breed	12	F	5	a
	DLBCL15	Cocker spaniel	5	F	3	a
	DLBCL16	Mixed breed	4	F	3	a
	DLBCL17	Mixed breed	6	F	4	a
	DLBCL18	German shepherd	8	F	3	a
	DLBCL19	Mixed breed	8	M	5	a
	DLBCL20	Mixed breed	5	F	4	a
	DLBCL21	Mixed breed	5	M	4	a
	DLBCL22	Mixed breed	11	F	5	b

Samples were preserved in RNA-later and stored at minus 80°C prior to RNA and DNA extraction. For all samples, DNA extraction was conducted using the DNeasy Blood & Tissue Kit (Qiagen, Hilden, Germany) according to the manufacturer’s specifications. Total RNA was extracted using the RNAeasy Mini Kit (Qiagen) according to manufacturer’s specifications. For each sample, RNA integrity and quality were estimated on the Agilent 2100 Bioanalyzer (Agilent Technologies, Palo Alto, CA). DNA and RNA concentrations were determined with a NanoDrop ND-1000 UV-Vis spectrophotometer (NanoDrop Technologies, Wilmington, USA).

### In silico Prediction of TFPI-2 CpG Island and Primer Design

In order to identify putative CpG island on promoter region, TFPI-2 genomic sequence (ENSCAFG00000002040), as stored on Ensembl genome browser (http://www.ensembl.org/index.html), including 1000 bp upstream the ATG site was employed as query sequence. Two different softwares, CpGPlot software (http://www.ebi.ac.uk/Tools/seqstats/emboss_cpgplot/) and MethylPrimer Express v1.0 (Applied Biosystem) were applied. Parameters set for CpG island identification were: 1) minimun length of 200 nt, 2) CG content ≥50% and, 3) CpG observed/CpG expected ratio ≥0.6.

On the CpG island identified, Bisulfite-specific primers (BSPs) were then designed by means of Methyl Primer Express software to amplify a region of 493 bp (see Table 2). In the same way, a second primer pair was designed as “template specific portion” of Fusion primers (see below) employed for 454 amplicon library preparation (see Table 2).

**Table 2:** Primer pairs employed for PCR amplification and amplicon library preparation.

	Primer	Primer sequence	Amplicon (bp)
**First-round PCR**	TFPI2_2F	GGGGAGGTAGGTTTAATTTGG	493
	TFPI2_Rd	TCCTAAACRCCCTATACAACTAAAA	
**Amplicon library preparation**	TFPI2_LF	GTGTGGGGTATGAATTAGTTAGG	212
	TFPI2_LR	AACCAACRAAATCCATAC	

### Bisulfite Treatment and PCR Amplification

For each sample, a total of 400 ng of genomic DNA was bisulfite treated using the MethylCode Bisulfite Conversion Kit (Invitrogen, Carlsbad, California) following manufacturer’s specifications. A total of 10 ng of bisulfite-converted DNA was then employed as template for first-round PCR using TFPI2_2F and TFPI2_Rd primers (see Table 2) in order to amplify a portion of 493 bp of TFPI-2 promoter. Cycling conditions were: initial incubation at 94°C for 2 min followed by 35 cycles at 94°C for 20 s, 61.7°C for 30 s and 72°C for 1 min. A final extension step at 72°C for 5 min was added at the end of the last cycle. Amplification products for each sample were then employed as template for amplicon library preparation.

### Amplicon Library Preparation and GS Junior 454 Sequencing

The amplicon library was purified using Agencourt AMPure XP beads (Beckman Coulter, Krefeld, Germany), quantified using the Quant-iT PicoGreen dsDNA kit (Invitrogen, Carlsbad, CA, USA) and equimolar pooled together for the emPCR (library at 1×10^7^ molecules/ul). All data were generated using GS Junior Sequencer Instrument software version 2.7 (Roche Applied Science, Mannheim, Germany). Image processing and amplicon pipeline analysis was performed using default settings of the GS RunBrowser software version 2.7 (Roche Applied Science).

### Gene Expression Analysis of TFPI-2

TFPI-2 expression levels were tested on 25 samples (7 healthy controls and 18 DLBCLs) by means of real-time RT-PCR. A total of 100 ng of RNA for each sample was reverse transcribed to cDNA using High Capacity cDNA Reverse Transcription Kit (Life Technologies). An aliquot (2.5 μl) of diluted (1∶50) cDNA template was amplified in a final volume of 10 μl, containing 5 μl Platinum SYBR Green qPCR SuperMix-UDG 2× (Invitrogen) and 0.25 μl of each gene-specific primer (10 μM). The amplification protocol consisted of an initial step of 2 min at 50°C and 2 min at 95° followed by 45 cycles of 10 s at 95°C and 30 s at 60°C. All experiments were carried out in a *LightCycler 480* thermocycler (Roche Diagnostics).

Each measurement was made in triplicate, and normalized to the reference gene, transmembrane BAX inhibitor motif containing 4 (TMBIM4), which was also measured in triplicate. TMBIM4 was chosen as reference in qRT-PCR assays since it was already tested in canine lymphomas by Aricò et al. [14] and did not show differences in its expression profile between healthy and pathologic samples.

To evaluate the efficiency of each assay (TFPI-2 and TMBIM4), standard curves were constructed amplifying two-fold serial dilutions (from 1∶20 up to 1∶640) of the same cDNA, which was used as calibrator. For both transcripts, efficiency of primer pairs was within the range 95 - 105%. For each sample, the Cp (Crossing point) was used to determine the relative amount of target gene.

### Data Analysis

The sets of reads from each sample, together with a “reference” genomic (not bisulfite converted) sequence, were analyzed by BiQ Analyzer HT software [15]. The methylation status of each read sequence was then determined based on a C to T conversion at each CpG. For each analyzed sample-reference combination, the software provides information about the methylation pattern *per* read and the mean methylation level calculated for the amplicon. In addition, a Pearl-Necklace diagram summarizes methylation information for the whole set of filtered reads, by CpG loci. For each site, distribution of the “methylated”, “unmethylated” and “unrecognized” states in the given set of bisulfite reads is also provided by the software.

The percentage of methylation (methylation level) at each CpG site within each amplicon was calculated based on the number of sequences containing methylated CpG sites versus the total number of sequences analyzed, assigned values range between 0 (0%, site unmethylated) and 1 (100%, site totally methylated).

Statistical analyses were performed using a commercially available statistical software program (SPSS v20.0). Data were analysed using non-parametric statistical methods because of the limited number of cases. Sample mean methylation levels, as well as methylation percentage of individual CpG sites, were evaluated for significant differences between healthy dogs and dogs with DLBCL using the Kolmogorov-Smirnov test.

Mann-Whitney test was performed on qPCR data to test for significant difference in TFPI-2 expression levels between controls and DLBCLs. Spearman’s rank test was used to estimate the correlation between mean methylation levels and TFPI-2 gene expression values measured for the same individuals. A p-value <0.05 was selected as the level of significance.

## Results

Twenty-two dogs with DLBCL were enrolled (Table 1). These were 15 females and 7 males, and the median age was 7,5 years (range, 3 to 14 years). Regarding breed, there were 11 mixed-breed, 2 German shepherds, 2 Labrador retrievers, and one each of Scottish terrier, Tibetian terrier, Pyrenean Sheepdog, Epagneul Breton, Yorkshire terrier, Cocker spaniel and English setter.

Nine healthy dogs were enrolled (Table 1): 5 mixed-breeds, 1 Siberian husky, 1 American Pit Bull terrier, 1 German shepherd, and 1 Cane Corso. Median age was 6 years (range, 2 to 10 years). There were 5 females and 4 males.

### TFPI-2 Amplicon Sequencing

**Figure 1:**
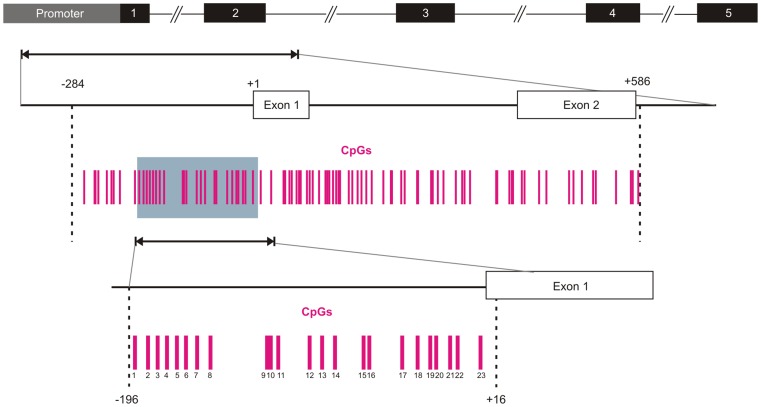
Schematic representation of of the TFPI-2 gene and CpG island. The black boxes indicate exons, +1 indicates the translation start site. Each vertical bar represents a CpG dinucleotide. From −284 to +586 is the location of the CpG island, the grey box (from −196 to +16) indicates the region analyzed by Bis-seq.

A region encompassing 870 nucleotides (−284 to +586) and containing 87 CG dinucleotides was identified as CpG island by means of CpG Plot software (Figure 1). A fragment of 212 nt, encompassing 23 CpG sites, was then chosen for ultradeep bisulfite sequencing analysis (see Methods).

A total of 14,755 sequences was obtained in a single 454 sequencing run with average read length of 229.97 bp. After quality filtering a total of 14,508 sequences (98.3%) was used for computing the mean methylation level for each sample. On average, 418 sequence reads (range of reads 236–869, including both forward and reverse strands) were obtained for each dog (see Table 3).

**Table 3:** TFPI-2 mean methylation level and total number of reads for each sample. Gene expression levels measured by means of qPCR are also reported.

	Sample	qPCR	Reads	Methylation level	Mean methylation
**Healthy ctrls**	Ctrl1	11.990	490	**0.093**	**0.141 (±0.08)**
	Ctrl2	1.336	459	**0.135**	
	Ctrl3	na	337	**0.121**	
	Ctrl4	na	273	**0.033**	
	Ctrl5	0.862	469	**0.308**	
	Ctrl6	1.290	349	**0.076**	
	Ctrl7	0.711	480	**0.158**	
	Ctrl8	1.563	427	**0.216**	
	Ctrl9	2.703	384	**0.128**	
**DLBCLs**	DLBCL1	0.030	410	**0.671**	**0.448 (±0.26)**
	DLBCL2	1.563	337	**0.931**	
	DLBCL3	4.636	364	**0.692**	
	DLBCL4	na	385	**0.863**	
	DLBCL5	0.184	485	**0.007**	
	DLBCL6	0.055	435	**0.133**	
	DLBCL7	0.031	441	**0.481**	
	DLBCL8	0.052	489	**0.395**	
	DLBCL9	1.441	331	**0.361**	
	DLBCL10	0.666	461	**0.082**	
	DLBCL11	1.324	236	**0.696**	
	DLBCL12	0.01	534	**0.552**	
	DLBCL13	0.000	402	**0.147**	
	DLBCL14	1.200	338	**0.889**	
	DLBCL15	0.114	352	**0.129**	
	DLBCL16	0.140	453	**0.324**	
	DLBCL17	na	374	**0.414**	
	DLBCL18	na	393	**0.382**	
	DLBCL19	2.264	457	**0.549**	
	DLBCL20	na	869	**0.306**	
	DLBCL21	1.546	397	**0.526**	
	DLBCL22	0.859	372	**0.316**	

### Quantitative DNA Methylation Analysis

For each sample, the mean methylation level (see Table 3) and the percentage of methylation for each CpG locus (reported in File S1) were calculated by means of BiQ Analyzer HT. Seventeen out of 22 DLBCLs (77.3%) exhibited a mean methylation ≥0.3, with 9 samples (40.9%) with level of methylation greater than 0.5. Control lymph nodes showed a mean methylation ≤0.3. Only one sample had a mean methylation of 0.31. Kolmogorov-Smirnov test was performed in order to compare methylation levels between controls and DLBCLs. A significant 3.2-fold increase of mean methylation levels in DLBCL dogs was observed compared to control lymph nodes (p<0.01). Five DLBCLs (i.e. DLBCL5, DLBCL6, DLBCL10, DLBCL13 and DLBCL15) did not show hypermethylation in TFPI-2 promoter, with mean methylation levels significantly reduced (p<0.01) compared to all other DLBCL samples.

### DNA Methylation Patterns on Individual CpG Sites

**Figure 2:**
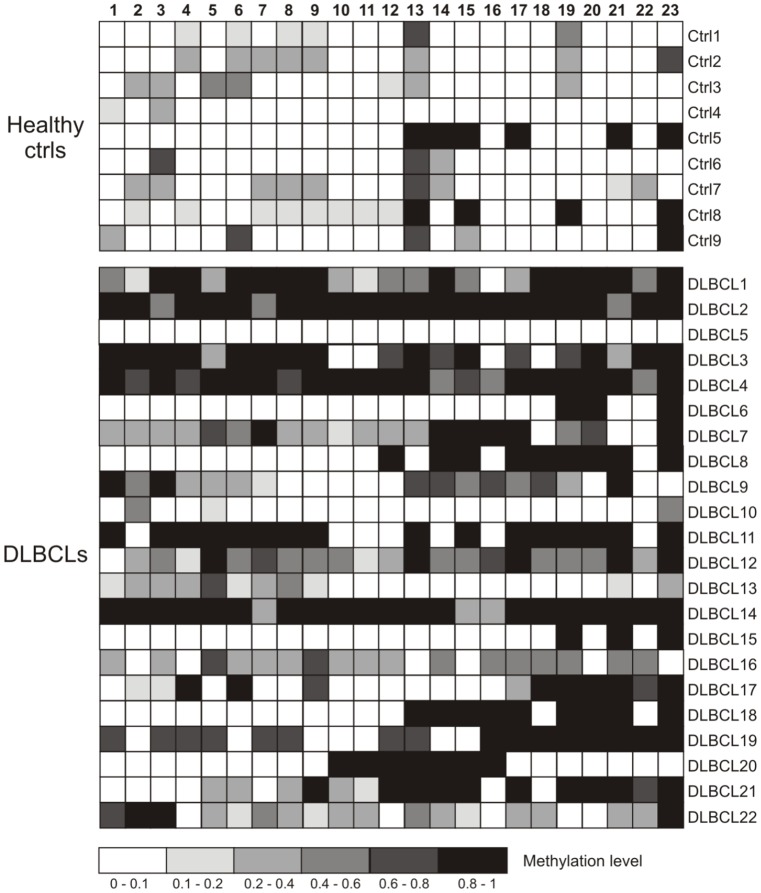
Bisulfite sequencing of the TFPI-2 CpG island. Each box indicates a CpG dinucleotide and each line of boxes represents analysis of a single dog. Color gradation indicated the methylation level of each CpG site (0–1); 0, totally unmethylated; 1 totally methylated.

When considering each CpG site individually (Figure 2), only one locus out of 23 showed a mean methylation percentage higher than 50% in healthy controls compared with 7 loci (30.4%) found in DLBCL samples (see File S1). The difference between the two groups was more evident if the threshold of the mean methylation percentage was lowered to 40% (0.4) with two loci (8.6%) and 15 loci (65.2%) for controls and DLBCL dogs, respectively. Statistical analysis for each CpG site identified eight loci with significant difference (p<0.05) in methylation level between DLBCL and control group (see Table 4). Excluding DLBCL5, DLBCL6, DLBCL10, DLBCL13 and DLBCL15 from the analysis, the number of loci found differentially methylated between the two groups increased up to 14 out of 23 (see Table 3).

**Table 4:** Kolmogorov-Smirnov test on 23 individuals TFPI-2 promoter CpG sites.

Site	p-value DLBCL n22	p-value DLBCL n17(a)
**CpG1**	0.082	0.014*
**CpG2**	0.517	0.410
**CpG3**	0.438	0.137
**CpG4**	0.143	0.034*
**CpG5**	0.016*	0.005**
**CpG6**	0.537	0.224
**CpG7**	0.17	0.058
**CpG8**	0.18	0.068
**CpG9**	0.143	0.034*
**CpG10**	0.138	0.031*
**CpG11**	0.622	0.239
**CpG12**	0.045*	0.006**
**CpG13**	0.537	0.410
**CpG14**	0.106	0.013*
**CpG15**	0.35	0.063
**CpG16**	0.143	0.031*
**CpG17**	0.016*	0.001**
**CpG18**	0.011*	0.002**
**CpG19**	0.042*	0.012*
**CpG20**	0.011*	0.002**
**CpG21**	0.007**	0.002**
**CpG22**	0.235	0.068
**CpG23**	0.031*	0.013*

(a) DLBCL5, DLBCL6, DLBCL10, DLBCL13 and DLBCL15 excluded from the analysis.

*p<0.05, **p<0.01.

### Relationship between TFPI-2 Methylation Status and Gene Expression Levels

**Figure 3:**
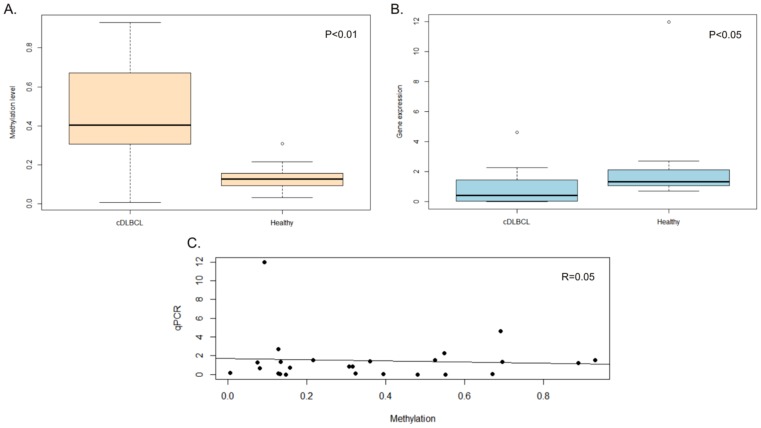
Relationship between TFPI-2 methylation and gene expression levels. **A.** Boxplot of TFPI-2 methylation levels measured in DLBCL and Healthy control group. **B.** Boxplot of TFPI-2 gene expression levels measured in the same samples and **C.** Correlation between TFPI-2 methylation and its gene expression values.

For a subset of samples including 7 controls and 18 DLBCLs (see Table 3), the association between TFPI-2 methylation levels and gene expression values was assessed by means of real-time RT-PCR (Figure 3). Gene expression analysis identified a significant down-regulation of TFPI-2 in DLBCL (p<0.05), with normal lymph nodes having 3-fold increased expression levels. Pearson correlation analysis between TFPI-2 methylation levels and gene expression, however, revealed a rho coefficient = 0.05 indicating that there is no linear correlation between the two variables (Figure 3).

Expression values obtained with qPCR were also correlated with those previously obtained using Canine genome 2.0 array (Affymetrix) [12] for the same specimens (n 19). Comparative analysis of microarray and qPCR expression data are reported in File S2. Statistical analysis identified a positive and highly significant correlation between qPCR and two microarray probes (Spearman’s 0.68≤ rho ≤0.78, p<0.01), thus validating microarray results.

The two oligonucleotide probes, Cfa.13994.1.S1_at and CfaAffx.3983.1.S1_at, showed a significant down-regulation of TFPI-2 in DLBCLs compared to control lymph nodes (p<0.05). However, as for qPCR, no linear correlation was documented between mean methylation levels and gene expression values.

### Relationship between TFPI-2 Methylation Status and Clinical Data

The Spearman’s correlations between TFPI-2 methylation levels, age, clinical signs, stage and substage were also assessed for all samples. Statistical analysis identified a significant positive correlation (Spearman’s rho 0.745, p<0.01) between methylation levels and age of DLBCL dogs. The number of dogs older than 8 years in healthy control group was too low for properly assessing the correlation age/methylation in this group.

No correlation was observed between TFPI-2 methylation levels and staging of the disease (data not shown). The same analysis was conducted considering TFPI-2 mRNA levels and no correlation was found between TFPI-2 expression and age or staging.

## Discussion

DLBCL is the most common subtype of canine lymphoma [14], [16] and it shares many features with the human counterpart, including clinical presentation, biological behavior, tumor genetics and treatment response [1]. A genome-wide hypomethylation associated with a simultaneous promoter-associated CpG island hypermethylation is now considered a hallmark of cancer [17]. Recently, genome-scale studies reported that aberrant DNA methylation occurs at specific genomic locations [18], [19]. It is widely reported that TSGs are frequently hypermethylated in neoplastic cells. This epigenetic modification contributes to the suppression of their expression ([20] and references therein) and makes cancer cells acquire growth advantages. However, the role of the single gene aberrant DNA methylation in controlling gene expression and tumor progression is still under debate.

TFPI-2 has been recently proposed as TSG since it plays a significant role in the maintenance of the stability of the tumor microenvironment and in the prevention of cancer progression by inhibiting tumor-related angiogenesis and members of ECM.

In particular, TFPI-2 has been reported to inhibit MMP-1, MMP-2, MMP3 and MMP-9 [21]–[23], proteolytic ECM enzymes whose increased expression has been demonstrated to be strongly associated with prognosis in canine lymphoma [14], [24].

The transcriptional silencing of TFPI-2 by hypermethylation in the promoter region has been recently demonstrated in many human cancers, including nasopharyngeal, colorectal and gastric carcinoma [9]–[11], [25]. No studies report TFPI-2 methylation in human lymphoma, although this phenomenon is described in pediatric acute myeloid leukemia [13].

In the present study, bisulfite conversion and 454 amplicon sequencing (Bis-seq) provided an accurate quantification of TFPI-2 methylation levels with a single base resolution, allowing the identification of CpG sites aberrantly methylated in canine DLBCLs. Sequence analysis clearly demonstrated that TFPI2 is frequently hypermethylated in canine DLBCL. Globally, dogs with DLBCL showed a mean methylation level over three-fold higher than controls and hypermethylation of the TFPI-2 promoter was found in 77% of DLBCLs (17 out of 22) against only one normal lymph node (∼11%). The analysis of hypermethylation by site identified 19 loci out of 23 (82%) with mean methylation levels from 2- to 120-fold increased in DLBCL (File S1) and 8 CpG loci were significantly (p<0.05) hypermethylated in DLBCL dogs compared to controls.

One of the proposed regulatory mechanisms ascribed to DNA methylation is to prevent the binding of transcription factors [26]. In dogs, TFPI-2 gene is located in chromosome 14 and, similarly to humans, *in silico* analysis of its promoter region identified the binding sites for several transcription factors, including SP1, activating enhancer-binding protein-1 (AP-1), nuclear factor kappa B (NF-kB), lymphoid transcription factor-1 (Lyf-1) and Myeloid zinc finger 1 (MZF-1).

Here, bisulfite sequencing results provided a partial representation of the potential control apparatus of TFPI-2. However, the region analyzed in the present study included both AP-1 and NF-kB putative binding sites. No CpG dinucleotides were located in close proximity to the AP-1 consensus sequence, while the region with the highest density of hypermethylated CpG sites (Sites 17–21, see Table 4) encompassed the NF-kB binding motif. It is well known that the transcription factor NF-κB controls a wide range of genes that are involved in multiple cellular processes, from apoptosis to proliferation and inflammation. In particular, Rel/NF-kB families of transcription factors are key regulators of B-cell development, proliferation and survival [27]. Human and canine DLBCLs are characterized by constitutive canonical NF-kB activity [28], [29] that promotes lymphomagenesis and chemotherapy resistance via overexpression of anti-apoptotic NF-kB target genes (i.e. Cyclin D1 and D2, Bcl-2). Very recently Mudaliar and co-workers [29], through the analysis of differentially expressed probe sets in canine and human DLBCL, found molecular signatures shared between dog and human. In particular, they reported the enrichment of the NF-kB signalling pathway in both species, with potential therapeutic implications, and further underscoring the dog as a translation model for human DLBCL.

In humans, mutations at either AP-1 or SP1 sites resulted in reduced TFPI-2 activity while NF-kB has not been demonstrated to directly control TFPI-2 transcription [30]. Whether TFPI-2 in dogs is controlled by the same mechanisms as in humans has not been assessed yet. However, the location of NF-kB binding motif in canine TFPI-2 promoter (i.e. immediately upstream of the ATG site) is different from humans, and it is unknown whether NF-kB exerts a role in regulating the transcription of this gene in dogs. In this context, it cannot be excluded that the hypermethylation of the NF-kB binding motif may exert a role in preventing the binding of this transcription factor.

The second aim of the present study was to assess the relationship between TFPI-2 methylation status and its gene expression levels measured by means of qRT-PCR. TFPI-2 showed a significant down-regulation in DLBCL compared with normal lymph nodes, suggesting that TFPI-2 hypermethylation negatively regulates its transcription.

In comparison with previous “semi-quantitative” approaches that mainly relied on the sequencing of 5–10 clones for measuring gene methylation levels, NGS allowed for a proper quantification of each CpG status. We decided then to verify whether a linear correlation between methylation levels and gene expression values could be determined.

No linear correlation was found between mean methylation levels and gene expression values. This was not unexpected, since a certain level of discordance between methylation and gene expression has been reported in both humans and dogs [31], [32]. In these cases, gene promoters showed mixed methylation patterns with regions completely methylated and regions unmethylated, the latter being responsible for the continued gene expression.

In the present study, the extremely high GC content of TFPI-2 promoter, and the approach chosen for amplicon sequencing (i.e. sequencing only one amplicon in favor of a greater number of sequences), limited bisulfite evaluation to one forth (212/870 nt) of the entire CpG island thus giving only partial information on the methylation levels of the promoter. Although a good indication of the relationship between TFPI-2 methylation and gene transcription has been achieved, the analysis of the entire promoter region is needed in order to properly investigate the correlation between methylation level and gene expression, as well as to identify potential critical, hypomethylated, regions that allow transcription in spite of global promoter hypermethylation or *vice versa*.

In this context, of particular interest are five DLBCL cases (DLBCL5, DLBCL6, DLBCL10, DLBCL13 and DLBCL15) that showed TFPI-2 transcription silencing even if the promoter regions appeared to be unmethylated (Table 3). In addition to the binding of transcription factors, other mechanisms, known to regulate gene transcription at different levels (i.e. recruitment of other members of the “transcription machinery”, interplay of enhancer molecules or small non-coding RNAs) may participate in controlling TFPI-2 mRNA levels thus leading to its silencing in DLBCL samples in spite of lower methylation levels.

Multiple changes such as decreased telomere length and altered gene expression have been linked to cellular aging. Recently, genomic hypomethylation and accumulation of CpG island hypermethylation has been associated with increasing age in humans [33]. Several genome-wide studies have revealed strong links between age-associated epigenetic changes and disease ([17] for a review). In particular, the hematopoietic stem cell decline in aging individuals was described as being determined by age-dependent changes in DNA methylation. To our knowledge, a similar phenomenon of accumulated promoter hypermethylation has not been demonstrated in dogs. In the present study, a significant positive correlation (rho 0.74, p<0.01) was appreciated between TFPI-2 methylation levels and age of DLBCL dogs.

This finding is in accordance with the study by Hannum and colleagues [33] where, by using a newly developed aging model based on whole genome DNA methylation, tumor tissue appears to be 40% older than the matched normal tissue from the same individual.

Although a higher number of samples both from normal dogs and dogs with DLBCL are needed, this study provides the first indication of age-associated epigenetic modifications in dogs.

To conclude, our results demonstrated that TFPI-2 promoter hypermethylation occurs frequently in DLBCL. TFPI-2 is also expressed at lower levels in neoplastic lymphoid cells compared to normal lymphoid cells, thus demonstrating an association between methylation and silencing of TFPI-2 expression.

Identifying aberrant methylated genes may provide a better understanding of the pathogenesis of canine DLBCL. The present analysis is far from showing a prognostic utility of TFPI-2 in cases of DLBCL. Further analysis will consider a set of canine lymphoma cell lines to test the efficacy of hypomethylating agents and a larger number of cases to assess whether TFPI-2 promoter hypermethylation may be considered in association with prognosis and therapy. In this context, novel gene therapy tools, including recombinant adenoviral-mediated TFPI-2 delivery, are currently being developed to regulate tumor growth providing potential novel therapeutic approaches for the treatment of DLBCL in dogs [34].

## Supporting Information

File S1 **Analysis of methylation status for each TFPI-2 CpG locus.**
(XLS)

File S2 **Comparative analysis of microarray and qPCR data.**
(DOC)
