# Supplementary material for: Epigenetic Silencing of TFPI-2 in Canine Diffuse Large B-Cell Lymphoma
Source: PLoS One. 2014 Apr 2;9(4):e92707. doi: 10.1371/journal.pone.0092707 (PMC3973630; doi:10.1371/journal.pone.0092707)
Supplement: File S2 — Comparative analysis of microarray and qPCR data. (DOC) [file pone.0092707.s002.doc]

**Comparison between microarray and qPCR in assessing TFPI-2 expression levels in canine DLBCL**

**Table S1** TFPI-2 expression levels assessed by two oligonucleotide probes, using Canine genome 2.0 array (Affimetrix), and by means of qRT-PCR

|  | **Sample** | **Cfa.13994.1.S1_at probe** | **CfaAffx.3983.1.S1_at probe** | **qPCR** |
| --- | --- | --- | --- | --- |
| **Healthy ctrls** | **Ctrl2** | 7.796 | 6.261 | 1.336 |
|  | **Ctrl5** | 5.500 | 4.332 | 0.862 |
|  | **Ctrl8** | 6.160 | 4.827 | 1.563 |
|  | **Ctrl6** | 5.576 | 4.901 | 1.290 |
| **DLBCLs** | **DLBCL5** | 3.842 | 3.546 | 0.184 |
|  | **DLBCL10** | 3.889 | 3.576 | 0.666 |
|  | **DLBCL15** | 2.916 | 3.635 | 0.1114 |
|  | **DLBCL6** | 3.786 | 3.434 | 0.055 |
|  | **DLBCL13** | 3.817 | 3.763 | 0.00 |
|  | **DLBCL16** | 3.586 | 3.517 | 0.140 |
|  | **DLBCL9** | 7.591 | 6.284 | 1.441 |
|  | **DLBCL8** | 3.803 | 4.105 | 0.052 |
|  | **DLBCL17** | 4.090 | 3.430 | na |
|  | **DLBCL7** | 3.383 | 3.700 | 0.031 |
|  | **DLBCL19** | 7.129 | 6.693 | 2.264 |
|  | **DLBCL12** | 3.059 | 3.677 | 0.01 |
|  | **DLBCL1** | 2.928 | 3.539 | 0.030 |
|  | **DLBCL3** | 5.807 | 4.607 | 4.636 |
|  | **DLBCL11** | 3.667 | 3.808 | 1.324 |

A Spearman rank-correlation test was used to assess the correlation between TFPI-2 expression values measured with RT-qPCR and microarrays, respectively, in a total of 18 experiments. High correlation coefficients between microarray probes and RT-qPCR were observed, with Spearman's rho 0.68 <rho<0.78 (p< 0.01), thus confirming a strong positive correlation between the two technologies.

**Table S2** Correlation between microarray and real-time RT-PCR expression data

|  | | | **Cfa.13994.1.S1_at** | **CfaAffx.3983.1.S1_at** | **qPCR** |
| --- | --- | --- | --- | --- | --- |
| **Spearman's**  **rho** | **Cfa.13994.1.S1_at** | Correlation coefficient | 1,000 | ,696^**^ | ,781^**^ |
|  |  | Sig. | . | ,001 | ,000 |
|  |  | N | 19 | 19 | 18 |
|  | **CfaAffx.3983.1.S1_at** | Correlation coefficient | ,696^**^ | 1,000 | ,680^**^ |
|  |  | Sig. | ,001 | . | ,002 |
|  |  | N | 19 | 19 | 18 |
|  | **qPCR** | Correlation coefficient | ,781^**^ | ,680^**^ | 1,000 |
|  |  | Sig. | ,000 | ,002 | . |
|  |  | N | 18 | 18 | 18 |
| **. p<0.01 | | | | | |

The two oligonucleotide probes, Cfa.13994.1.S1_at and CfaAffx.3983.1.S1_at, showed a significant down-regulation of TFPI-2 in DLBCLs compared to control lymph nodes (p<0.05). However, as for qPCR, no linear correlation was appreciated between mean methylation levels and gene expression values (Figure S1).


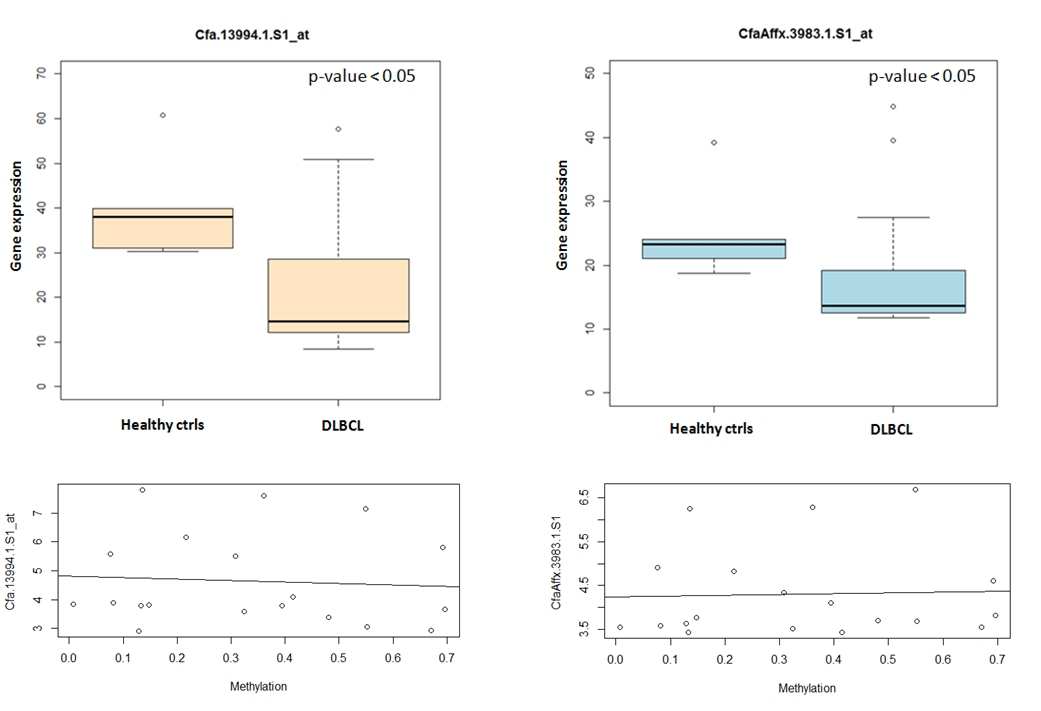


**Figure S1** TFPI2 expression values measured by oligonucleotide probes in Healthy ctrls and cDLBCLs (above) and their correlation with TFPI-2 promoter methylation levels (below).
